# Strabismic Adults' Expectations of Psychosocial Support From Healthcare Professionals–A Qualitative Descriptive Study

**DOI:** 10.1002/hsr2.70698

**Published:** 2025-05-04

**Authors:** Anna Mason, Katja Joronen, Laura Lindberg, Marika Kajander, Nina Fagerholm, Anja Rantanen

**Affiliations:** ^1^ Faculty of Social Sciences, Health Sciences Tampere University Tampere Finland; ^2^ HUS Helsinki University Hospital Head and Neck Center and Helsinki University Helsinki Finland; ^3^ Department of Nursing Science University of Turku Turku Finland

**Keywords:** health‐related quality of life, inductive content analysis, psychosocial support, qualitative research, strabismus

## Abstract

**Background and Aims:**

Strabismus, an eye misalignment, impacts the functional and psychosocial health‐related quality of life in adult patients. Corrective surgery generally improves adults' health‐related quality of life. Previous research reports that strabismic adults with a psychosocial influence of the condition may benefit from preoperative psychosocial support, although what such support should consist of is unclear. Therefore, this study aimed to describe strabismic adults' expectations of psychosocial support from healthcare professionals.

**Methods:**

The study has a qualitative descriptive design. It included 12 semi‐structured interviews with purposefully recruited patients who had experienced the psychosocial influences of strabismus. The data were analyzed by inductive content analysis.

**Results:**

Strabismic adult patients described their expectations for psychosocial support, consisting of education about strabismus, genuine encounters with strabismus experts, accessible support, and available peer support. The participants explained that strabismus specialists should provide tailored education for patients, the public, and other healthcare professionals. They shared that strabismus professionals should ask about patients' psychosocial well‐being—that by intently listening, openly discussing, and validating patients' experiences, they could support their well‐being. Psychological care provided by nurses and psychologists should be easily accessible, supportive, and timely for children and adolescents. Participants also expected professionals to guide them to available peer support.

**Conclusion:**

Strabismic adult patients dealing with the condition's psychosocial burden expect psychosocial support from strabismus experts. This study's results can be used to improve psychosocial care in clinical strabismus services.

## 1 Introduction

Strabismus is an eye condition in which the eyes are either misaligned (tropia) or a person has a latent strabismus (phoria). In manifest strabismus, the deviation can be constant, intermittent, or also alternate between the eyes [1, 2]. The worldwide estimated prevalence of strabismus in adults is 3%−4% [3], and horizontal deviations of exo‐ or esotropia are more common than vertical deviations of hypo‐ or hypertropia [2]. Strabismus in adults might be childhood‐onset or caused by other health conditions, for example, neurological diseases, thyroid eye disease, trauma, or vision loss [4]. The condition can be addressed surgically, with glasses, prism lenses, or botulinum toxin injections [2, 5].

Strabismus impacts one's health‐related quality of life (HRQOL) [6], which is defined as self‐reported well‐being and functioning in mental, physical, and social aspects of life [7]. Diplopia, ocular discomforts, eye strain, difficulties with depth perception, blurred vision, and headaches may impact functioning in everyday life [5, 8, 9]. Strabismus may also affect one's psychosocial functioning, for example, struggling in or even avoiding social situations, hiding one's strabismus, or averting one's gaze [6, 8, 9, 10, 11]. Symptoms of anxiety and depression and struggles with self‐perception, social bias, and one's appearance can decrease mental well‐being [5, 9, 12, 13, 14]. Corrective surgery generally improves patients' well‐being and HRQOL [5, 13, 15], which quality of life measures such as the Adult Strabismus Questionnaire (AS‐20) can evaluate [16, 17]. Some strabismic patients might require psychological or psychosocial support preoperatively to improve their postoperative well‐being [2, 12, 13]. However, evidence is lacking what psychosocial support for strabismic adults should consist of.

Psychosocial support, which includes both psychological and social support, is defined as activities and processes that improve one's comprehensive well‐being in their social environment [18]. Psychosocial support can also be given by family and friends [18]. In healthcare, psychosocial support means supporting patients and their families with emotional, mental, social, spiritual, and informational needs [19, 20]. Psychosocial support for patients with ophthalmic conditions can include informational, social, psychological, and peer support. Patients can benefit from informational support, such as medical education and information on treatment options [21, 22, 23]. Social support by other people can help patients adjust to new conditions or help with practical daily tasks [24, 25]. Psychological support, such as meditation, stress reduction techniques, or counseling with healthcare professionals can aid well‐being and treatment adherence of the patients [22, 26, 27]. Peers supported patients with ophthalmic conditions (e.g., glaucoma) to help them understand the illness and receive hope regarding the treatment [21]. As the knowledge of psychosocial support and what it should include is limited for strabismic adults, this study aimed to describe strabismic adults' expectations of psychosocial support from healthcare professionals.

## 2 Methods

### 2.1 Study Design and Settings

This qualitative descriptive study, which used semi‐structured interviews and inductive content analysis, was conducted in a Finnish university hospital's ophthalmology clinic that cares for patients with strabismus. Over 3000 outpatient visits and 350 surgeries occurred in 2022. A qualitative research design was chosen to explore and understand patients' expectations of psychosocial support [28, 29]. Standards for Reporting Qualitative Research (SRQR) checklist guided the reporting of the study [30].

### 2.2 Ethical Considerations

The tenets of the Helsinki Declaration and national research guidelines were followed [31, 32]. The Committee on Medical Research Ethics (HUS/3264/2021) and Head and Neck Center at HUS Helsinki University Hospital (HUS/148/2022) approved the study. All participants received written and verbal information about the study and signed informed consent to confirm their participation. According to the organizational data policies, patient confidentiality and data security were always maintained.

### 2.3 Participants

Multidisciplinary staff at the clinic purposefully recruited patients with psychosocial symptoms of strabismus and who were willing to describe their experiences and expectations to recruit individuals with the most insight into the studied phenomenon [33]. Inclusion criteria consisted of adult patients (≥ 18 years) who had sought treatment for strabismus and experienced psychosocial symptoms from the condition. Patients had to manage communication in Finnish and have no severe neurological or psychiatric health conditions. Potential participants were identified at the appointment by their answers on the Finnish Adult Strabismus Questionnaire (AS‐20) [17, 34]. Participation was offered to patients who had answered *sometimes, often, or always* on the Finnish AS‐20 items 1−10 measuring psychosocial HRQOL. Participation was voluntary and did not impact care. Before interviews all participants had seen an orthoptist or an optometrist on the current visit. They had seen an ophthalmologist either on the current or a prior visit. As per clinical care process, prior the first visit, all patients received a written information leaflet which include etiology of strabismus, options for correction, and the care process in the unit. In the leaflet it is highlighted that the care for strabismus is individual, and the need will be assessed in the appointment. It has been noted in clinical care that giving written information prior to the first visit gives patients or their parents opportunities to formulate questions beforehand. Altogether, 18 patients were approached for participation, 13 of whom signed an informed consent. Five declined due to a lack of interest or schedule conflicts. One patient did not respond to several interview requests; therefore, 12 adult patients were interviewed individually. Eight interviews were conducted face‐to‐face in a private room after the appointment, and four participants chose online interviews on Microsoft Teams. No other people were present during the face‐to‐face interviews, and online interviews were conducted in a private space so that no one else could see or hear the meetings. Participants allowed audio‐recording and permission was also received for the video recording of three online interviews. The participants were asked about two themes: first, their experiences of the psychosocial influence of strabismus and, second, their expectations of psychosocial support from healthcare professionals. This article reports the second theme; the first theme is reported previously [11].

### 2.4 Data Collection

The first author, a doctoral researcher in nursing science, conducted the interviews from August 2022 to February 2023. A semi‐structured interview guide was used to guide the interviews [28]. The guide was based on previous research on the psychosocial influence of strabismus and was produced jointly with the research group. Before the interviews commenced, the first author explained the interview process, research objective, confidentiality, data security, and the reasons for studying patients with strabismus. She also confirmed voluntary participation and informed consent; participants were encouraged to ask questions. She shared that although she has a nursing background, she was not a clinical expert on strabismus and did not participate in caring for strabismic patients. However, her work experience in ophthalmology has led to an interest in HRQOL among strabismic patients, and the study results could help improve multidisciplinary health services and psychosocial care for strabismic adults. All patients were informed of their right to a psychologist appointment if they felt the need after sharing their experiences.

Participants were given ID codes and asked for their sex and age, which was categorized to maintain anonymity. The onset of strabismus, previous strabismus surgeries, the appointment type, and whether misalignment was in one or both eyes were enquired for background questions. Participants were first asked about their experiences of the psychosocial influence of strabismus and then their expectations for psychosocial support. Exploratory questions were used to check for the interviewer's understanding of the participants' expectations [28]. With participants' permission, field notes of nonverbal communication, such as “crying when talking of the experience” or “seems happy to describe,” were recorded during the interviews to understand participant meanings [33, 35]. Toward the end of the interview, the interviewer checked that all themes in the interview guide were covered and asked the participants to share anything else relevant to the topic. The interviews lasted from 28 to 45 min, and the patients were interviewed once. Data analysis was conducted at the same time with data collection and the data for this study question was saturated after 11 interviews as no new information was gathered [29, 33]. However, one more interview was conducted to confirm the saturation.

### 2.5 Data Analysis

Inductive content analysis was chosen as the data analysis approach for a deeper understanding of patients' experiences and expectations, as the previous knowledge is deficient in this patient group [36, 37]. The method is data‐driven and content‐sensitive and aims to abstract raw data into categories which answer the research question [37]. The analysis process followed Kyngäs et al.'s phases of data reduction, data grouping, and the formation of concepts. First, the data was prepared by transcribing recordings after the interviews and listening to them several times to ensure the transcriptions were authentic. Field notes were recorded in transcriptions as latent content. Next, the first author carefully read the transcriptions with the following research question in mind: “What kind of psychosocial support do strabismic adults expect from healthcare professionals?” This was done to get an overview of the participants' expectations. She then highlighted the transcribed text units of analysis, which were meaningful sentences for the research question. Participants' ID codes were added to the units of analysis to enable her to return to the transcribed text and ensure a correct understanding of the expectations. Units of analysis were further reduced into open codes of words or short sentences to keep essential data for the research question. She was very aware of not making subjective interpretations on data and maintained a clear connection between the open codes and raw data, as is required in inductive content analysis [37]. Several units of analysis produced more than one open code. The abstraction process continued by comparing open codes for differences and similarities. Similar codes were grouped to create subcategories, which were given descriptive names. Similar subcategories were then further abstracted to form categories describing the strabismic adults' expectations for psychosocial support from healthcare professionals [36, 37]. Although the first author conducted the analysis independently and manually on Microsoft Excel, the research group discussed and reviewed it at all stages to ensure the trustworthiness of the analysis [38, 39]. Table 1 presents an example of the analysis to aid the reader in following the analysis.

**Table 1:** Example of the analytical process [37].

Units of analysis^a^	Open codes	Subcategory	Category
I feel the patient leaflets are good, but maybe it would be good to add where to find more information on strabismus. ID 11	Sources for further information should be included in patient leaflets. ID 11	Patient education	Education about strabismus
It would be important to have an information packet written with compassion. There are, of course, medical libraries online that are accessible to all, but the information is written in the medical style. ID 19	The information is written with compassion. ID 19		
I feel that strabismus should be talked about more so other people—the public—would know about it. ID 20	Strabismus needs to be communicated to the public. ID 20	Public education	
Strabismus is not shown anywhere, and it is unsurprising that no one knows anything about it. ID 2	Strabismus is generally unknown. ID 2		
It helps when one can talk about their feelings and struggles, and the professional focuses on listening. ID 4	Focused listening helps the patient. ID 4	Intently listening to the patient	Genuine encounters with strabismus experts
When I look back 10 years ago, if someone would have asked how I am, I would have felt heard. That kind of experience would have been important—understanding there are other problems than just the physical problem. ID 19	The experience of being heard is important. ID 19		
Professionals do not always have the courage to approach some subjects—like psychological well‐being. There should be courage because patients rarely talk about these things if not asked. ID 10	Professionals should bravely ask about one's psychological well‐being. ID 10	Bravely enquiring about psychosocial well‐being	
You can always ask if someone has had mental or social struggles. Many patients will not talk about them nor ask for help if it is not offered. ID 13	Professionals should ask about psychological and social struggles. ID 13		

^a^ Several units of analysis produced more than one open code.

## 3 Results

Twelve adults with manifest strabismus participated in the study. All had been referred for treatment to the unit. Eight were female, and five reported their age category as 18–30. Nine had previous strabismus surgery as they had childhood‐onset strabismus. All had experienced the influence of strabismus on psychosocial well‐being and felt that support should be offered. Four patients spontaneously reported functional symptoms of diplopia, and the other four described difficulties with eye strain, headaches, and torticollis. Two of the participants were on the first visit at the unit and two patients had preoperative appointments. Majority (*n* = 11) of the participants were listed for surgery, although due to the length of waiting time, they attended follow‐up appointments for reassessment of their condition. Table 2, which was published before without the types of visits, presents the participating patient characteristics [11].

**Table 2:** Participants' characteristics [11].

Participant ID	Sex	Age categorized	Previous surgery	Onset of strabismus	Type of current visit	Interview method	Duration (in min)
1	Female	18–30	1	Childhood	Follow‐up visit	Online	35
2	Female	64−	0	Adult	First visit, no prior visits	Face‐to‐face	44
3	Male	45–63	3	Childhood	First visit for this referral, awaiting surgery	Online	40
4	Female	64–	0	Adult	First visit, no prior visits	Face‐to‐face	38
8	Female	45–63	1	Childhood	Follow‐up visit	Face‐to‐face	34
9	Male	31–44	1	Childhood	Follow‐up visit	Face‐to‐face	28
10	Male	45–63	0	Adult	Follow‐up visit	Face‐to‐face	34
11	Male	18–30	2	Childhood	Preoperative visit	Face‐to‐face	36
12	Female	18–30	2	Childhood	Follow‐up visit	Online	36
13	Female	18–30	3	Childhood	Preoperative visit	Face‐to‐face	35
19	Female	31–44	2	Childhood	First visit for this referral	Online	45
20	Female	18–30	1	Childhood	Follow‐up visit	Face‐to‐face	44

**Figure 1:**
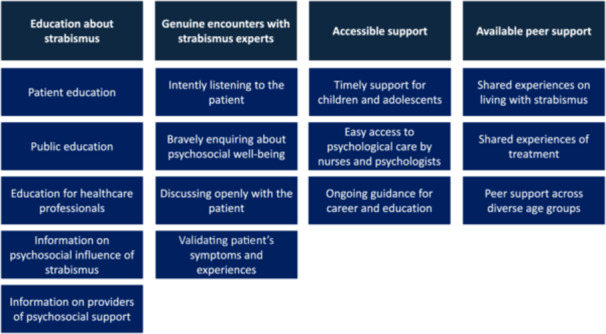
Strabismic adult patients' expectations for psychosocial support.

Participants expressed their expectations for psychosocial support as the need for education about strabismus, genuine encounters with strabismus experts, accessible support, and available peer support. They also recommended regular use of an HRQOL measure, such as AS‐20, in all clinical strabismus services to assess the condition's impacts. They explained that AS‐20 could be used as a tool to encourage the professionals to ask about psychosocial well‐being during the appointment. Figure 1 describes the results, and the participants' authentic quotations are presented with the results.

### 3.1 Education About Strabismus

Patients were pleased with the verbal and written information given by the unit. They expected to receive understandable patient education from strabismus specialists, including etiology, possible treatment options, and how the care is planned. Additionally, they wanted to understand how aging impacts strabismus and what might happen if strabismus is untreated. They explained it is important to know if treatment would be beneficial, to understand their symptoms, and to receive explanations about what happens during surgery. The patients felt they received this information in the unit. However, some patients expected additional information using medical terms, provision of further information sources, or research explained in layperson's language. They explained that receiving adequate information reduces calls afterward with questions. They highlighted that, if they had questions, they wanted direct access to the professionals who treated them.

Well, maybe the support could be information – of all the possibilities to treat and correct strabismus so that all options would be presented: what could be done in various situations and how strabismus could be corrected and treated.

ID 3

Participants wished that strabismus specialists would be active in sharing general information about strabismus to the public at all ages. They explained that because strabismus is unknown in their social circles or generally in society, they had to regularly explain to others what strabismus is and how it influences their HRQOL. They hoped experts would use colloquial language and publicly speak about the condition whenever possible.

Always tell others about strabismus when you have a chance to talk about it.

ID 20

They described difficulty accessing strabismus care as general physicians, ophthalmologists, or school nurses did not understand their condition or the available treatment and care pathways. They hoped that strabismus specialists would educate other healthcare professionals on strabismus and its treatments, and the increased knowledge could improve access to care and, therefore, provide support.

It feels like the general doctors, who are not specialized in strabismus, just do not understand this condition.

ID 12

Interviewees hoped to receive information on their appointments from strabismus experts on the condition's psychosocial influence. The experts should inform them that strabismus may have a psychosocial impact on well‐being and that one is not alone in experiencing it. Information on providers of psychosocial support was also expected.

Well, it could be… during the assessment and maybe even something to take home – not to just talk about symptoms or generally about strabismus but what kind of feelings or thoughts the patient might have.

ID 10

It would be lovely if… you could say this (support) is what we have.

ID 13

### 3.2 Genuine Encounters With Strabismus Experts

The participants described their experiences of appointments with strabismus experts as places where they had been genuinely encountered. They had experienced that when they were intently listened to, they were already supported.

I do not want my issues or symptoms to be underestimated; instead, I need to be listened to when I talk about my issues and symptoms with strabismus and so on… I feel it has been the best support I have received so far.

ID 12

To improve the encounters at the appointments, patients shared that strabismus experts should bravely ask about their patients' psychological and social well‐being and not be afraid to approach the subject. It would be important to ask how strabismus influences the patient's life and well‐being and whether they need help to go forward and someone to talk to.

By listening, asking how they have been… do they need support and someone to talk to go forward with their life?

ID 4

The adults also described that open discussion between strabismus professionals and their patients was important. Such dialogue could help patients relax during the appointment, increase their well‐being, and improve their self‐esteem.

Open discussion could help with self‐esteem, too.

ID 9

Participants felt that receiving validation for their symptoms and experiences from strabismus experts at the appointment was important. For example, their experiences are real; they are not the only ones who feel this way, and so forth.

I would say that healthcare professionals can validate these experiences and issues you have dealt with. You are not alone in them. Others have very similar feelings and the same experiences.

ID 12

### 3.3 Accessible Support

Participants expected psychosocial support to be accessible. As the participants with childhood‐onset strabismus reflected on their childhood and adolescence, they expected support to be timely for children and adolescents so that young patients did not have to wait until adulthood to receive it.

Hmm, already in childhood, someone should have done something. I feel sad looking back, knowing I did not get any professional support as a child. There should have been help with strabismus then. There should have been a place where I could have talked about how I feel about my eyes.

ID 19

Patients described the need for easy access to psychological care by nurses and psychologists. The support should be regular practice and available as required; the professional should also have knowledge of strabismus. The participants suggested that a psychiatric nurse could be based in the unit for easy access to discuss experiences, negative emotions, and the impacts on psychosocial well‐being. They felt a psychologist could help them cope with strabismus without thinking about it constantly. Both nurses and psychologists could provide support to help them cope with anxiety or stress due to strabismus and offer practical strategies on how to talk about the condition with others. A face‐to‐face appointment with a nurse could be a place to practice keeping eye contact, as strabismic adults often avoid eye contact in interactions. If required, the professional could refer the patient to further therapy services.

For me, it is important to speak… I get to speak my own thoughts, and a psychologist could then throw back some ideas about how I could think differently or live with strabismus.

ID 11

Participants hoped psychosocial support would include ongoing guidance with a career specialist in choosing a profession. This was highlighted by the participants whose strabismus was difficult to treat and whose functional symptoms restricted their employment, which, together, caused emotional symptoms. The specialist having knowledge of strabismus would be imperative.

The support could also have some kind of career counseling or similar services… what occupation and education could be chosen if symptoms are difficult to treat.

ID 8

### 3.4 Available Peer Support

The participating patients shared that peer support is necessary and expected as part of psychosocial support. They were realistic in that healthcare professionals could not organize peer support but could guide patients in the right direction. Participants expressed that meeting others with strabismus would be beneficial and give them an opportunity to discuss how others have coped and overcome the challenges associated with strabismus. Receiving emotional support and sharing the disease burden would be easier with others with strabismus rather than their non‐strabismic peers. Others with the same condition would understand their challenges. They felt that seeing others with strabismus and sharing their experiences was important.

Well, I feel it is very simple: When people with strabismus meet up, that already helps and supports you, as you see that there are others [like you]; you are not alone.

ID 2

The participants expressed that hearing others share their experiences of treatment and surgery could be beneficial. Knowing whether others found surgery useful and how it helped them would be helpful.

I think if people are considering surgery and whether it would be beneficial, it would be good to hear how others have experienced it. It would be good to offer peer support.

ID 9

As strabismus affects people of all ages and backgrounds, the participants suggested that peer support should not be limited by age or background. People of different ages and backgrounds could offer new perspectives and priorities when sharing their experiences.

Yes. And if the peer support group has people of different ages, with different life situations and experiences, and they come from all over… Have you thought this? There is also this side: Maybe it could help and make people think more and put their priorities right…

ID 10

## 4 Discussion

This study aimed to describe strabismic adults' expectations of psychosocial support from healthcare professionals. Patients reflected on the support they had received and the support they would expect. Strabismic patients with psychosocial symptoms struggle with their well‐being [9, 12, 14], and psychosocial support is recommended [2, 12]. However, the previous knowledge of strabismic adult's expectations and the content for psychosocial support is lacking. Therefore, this study results can aid to fill knowledge gap on the phenomenon. Psychosocial support should be provided regularly for patients who require it, be accessible, and include education about strabismus, genuine encounters with strabismus experts, and available peer support.

Participants described a need for patient education on strabismus, its psychosocial influences, and available support. They felt that education on strabismus given to them had been useful although they would have expected more information on psychosocial symptoms and providers on psychosocial support. This concurs with previous evidence of how clear and timely information on the condition, treatment options, and prognosis can support patient's well‐being and encourage them to become more involved in their care [21, 23]. However, it is unclear what the golden standard for patient education for strabismic adults should be as many factors can influence a patient's ability to learn and accept information [40]. In current clinical practice, the patients are sent an information packet prior their first visit and this has helped preparation for the appointment. This practice can enhance patient‐centered care as intensive patient education is one of the methods for improving patient‐centered care in ophthalmology [41]. As nearly all the participants in the current study were listed for surgery, patient‐centered care could be further advanced by introducing the Expectations of Strabismus Surgery Questionnaire (ESSQ) for understanding patient needs more [42]. Additionally, majority of the participants had childhood‐onset strabismus and previous surgeries indicating previous knowledge of their condition and this could have influenced the results. It is notable that the ability to communicate in Finnish was one of the inclusion criteria in the current study. Patients in the unit come from different backgrounds and languages, and it is possible that their health literacy levels are not as good as the current study participants. Therefore, in patient education, health literacy should always be considered [40].

Patients felt that strabismus is not well known in public or generally among healthcare professionals; therefore, education is needed. Our study highlighted public education in the context of increasing societal awareness of strabismus to support patients' psychosocial well‐being. Other studies have shown the importance of public education, which can raise awareness of the condition, thus reducing delaying surgery [4, 43]. Education is also required for healthcare professionals in all levels of healthcare as their lack of knowledge in strabismus hindered this study participants' access to care. This result is similar to previous findings [4, 5, 43].

Participants expected genuine encounters with strabismus experts, such as bravely asking about the psychosocial influences of strabismus, having open discussions, and receiving validation for their symptoms. They also described their need to be listened to. It is necessary for the clinicians to approach and recognize patients' psychosocial symptoms preoperatively to improve postoperative outcomes [12]. Knowledge of a patient's emotional well‐being might also change how the professional communicates with the patient and improve any referrals to mental health and social services [44]. Using open communication, such as open rather than close‐ended questions, and listening to the patients can improve the overall patient‐centered care [41], the patient experiences [21], and quality of the care [45]. The study in patients with chronic pain concluded that validation of symptoms and experiences can reduce psychosocial stress and act as a protective factor for patients' psychosocial health [46].

The current study participants expected accessible support, timely for children and adolescents, easy access to psychological care by nurses and psychologists and ongoing guidance with a career specialist. Healthcare professionals should be aware of the psychological burden of strabismus among children and refer them for support accordingly [47]. Accessible psychosocial support is imperative as studies by McBain et al. [14] and Adams et al. [12] reported that strabismic patients had symptoms of depression and clinical anxiety more often than the general population. Psychological care by psychologists or nurses could utilize different stress reduction techniques, meditation or counseling [26, 27], or psychological nursing which have shown to improve postoperative psychological well‐being in glaucoma patients [48].

Peer support was considered an integral part of psychosocial support in this study. Patients felt they could share their experiences and struggles with other people with strabismus, who would understand how they felt. Sharing experiences on surgery and other treatments would benefit others with strabismus. This is consistent with a study on self‐management challenges and support needs in patients with glaucoma [21].

Results should also be evaluated from the perspectives of resources, logistics, and feasibility. Some of these expectations for support require resources in the current clinical practices and, therefore, might not be implemented. Virtual healthcare, such as a Digital Care Pathway [49] especially with audio‐visual material could be a logistical and feasible solution in providing psychosocial support for strabismic patients including those with functional symptoms [5, 8]. Most of the patients in the study were listed for corrective surgery which is shown to improve psychosocial health postoperatively [12, 13, 50], and their expectations for support could have been different if interviewed postoperatively. Therefore, more information is needed on the impact of surgical alignment in Finnish population prior developing psychosocial support intervention.

### 4.1 Strengths and Limitations

The trustworthiness of this qualitative study can be assessed by the criteria of credibility, transferability, dependability, and confirmability [39]. Credibility was enhanced by purposively recruiting patients who had struggled with the psychosocial influences of strabismus and were willing to talk about their expectations for psychosocial support. The participants represented different ages, sexes, and childhood‐ and adult‐onset strabismus. Inclusion and exclusion criteria, data collection and analysis, and patient demographics were reported clearly. An example of the analysis process is provided, and participants' authentic quotations improved trustworthiness. All these strengthen the credibility, transferability, and dependability of the study results. The research team discussed the study in all stages, supporting the self‐awareness and self‐reflection of the first author. She returned several times to the transcribed text to ensure the abstraction process was true to participants' expectations, enhancing the study's confirmability.

This study has limitations. Notably, the participants were 12 adults with manifest strabismus who sought help for their condition and had psychosocial influences of strabismus. Therefore, the results are not transferable to all strabismic adults or patients with other ocular conditions. We did not ask the patients their socio‐demographic data, except the age, or did not have access to the patients' clinical strabismus data, such as the deviation or the angle of strabismus and therefore, we cannot describe the patients' clinical background factors. Obtaining these could have provided a deeper understanding of expectations between patients with different education levels, types of strabismus, symptoms, deviation direction, or size. The lack of information decreases transferability and limits the study. Additionally, recruiting the participants based on AS‐20 responses potentially introduced bias, as patients who had received psychosocial support and their AS‐20 scores had improved due to support, were excluded. It is notable that the participants were waitlisted for strabismus surgery, and the length of waiting time could have influenced their expectations for psychosocial support and thus impacted the results of this study. Dependability is decreased as participating patients did not evaluate study findings. Future studies should observe these factors.

## 5 Conclusions and Implications for Clinical Practice

This study described strabismic adult patients' expectations for psychosocial support, which consisted of education about strabismus, genuine encounters with healthcare professionals, accessible support, and available peer support. Participating patients were recommended to use an HRQOL measure to assess well‐being. Healthcare professionals and researchers specializing in strabismus should educate the public and other healthcare professionals on strabismus, its treatments, and its impact on one's HRQOL to aid the psychosocial well‐being of the patients and easier access to care.

The results of this study can be used to improve psychosocial care in clinical strabismus services. Virtual healthcare could be utilized in development of psychosocial support although more information is needed to understand how corrective surgery influences psychosocial health.

## Author Contributions

**Anna Mason:** conceptualization, data curation, formal analysis, funding acquisition, investigation, methodology, project administration, visualization, writing – original draft, writing – review and editing. **Katja Joronen:** conceptualization, methodology, supervision, validation, writing – review and editing. **Laura Lindberg:** conceptualization, validation, writing – review and editing, resources. **Marika Kajander:** validation, writing – review and editing, conceptualization. **Nina Fagerholm:** conceptualization, funding acquisition, writing – review and editing, validation, resources. **Anja Rantanen:** conceptualization, funding acquisition, methodology, supervision, validation, writing – review and editing. All authors have read and approved the final version of the manuscript.

## Conflicts of Interest

The authors declare no conflicts of interest.

## Transparency Statement

The lead author, Anna Mason, affirms that this manuscript is an honest, accurate, and transparent account of the study being reported, that no important aspects of the study have been omitted, and that any discrepancies from the study as planned (and if relevant, registered) have been explained.

## Data Availability

Data cannot be shared due to the ethical approval of the study. Anna Mason had full access to all of the data in this study and takes complete responsibility for the integrity of the data and the accuracy of the data analysis.
